# Double drainage of total anomalous pulmonary venous connection revealed after surgical repair of a supracardiac total anomalous pulmonary venous connection

**DOI:** 10.1002/ccr3.3352

**Published:** 2020-09-15

**Authors:** Hiroyuki Nagao, Kenta Tominaga, Naoya Kamei, Toshikatsu Tanaka

**Affiliations:** ^1^ Department of Cardiology Hyogo prefectural Kobe children's hospital Kobe Japan

**Keywords:** catheterization, coil embolization, congenital heart disease, mixed total anomalous pulmonary venous connection, veno‐venous shunt

## Abstract

Mixed type TAPVC with double drainage, where the second vertical vein enlarged rather than atrophied after surgery. If we did not recognize left‐to‐right shunting before surgery, it can be treated with catheterization as demonstrated by our case.

## Graphical abstract

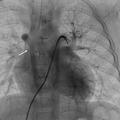

## 1 INTRODUCTION

Total anomalous pulmonary venous connection (TAPVC) is an anomaly where the pulmonary veins are directly connected to one of the systemic veins or to the right atrium. The most common classification of this condition is by Craig, Darling, and Rothney, which is based on the anatomic site of the abnormal connection. A few variants are reported which do not fit into these classic forms.

We report 2 cases that previously underwent surgical repair of supracardiac TAPVC, where the pulmonary veins drained into the innominate vein through an ascending vertical vein. However, these cases did not fit into the classic forms, because in both cases, a second vertical vein was detected incidentally during a follow‐up diagnostic catheterization 1 year after the initial surgery. The second veins originated proximal to the ligated vertical vein and drained into the superior vena cava through the accessory hemiazygos vein and azygos vein.

In Case 1, which was did not provoke right heart volume overload and general condition is good. The patient is followed up carefully, even in the absence of any intervention or surgical intervention.

In Case 2, we confirmed right heart volume overload and then performed coil embolization of the veno‐venous shunt (VV shunt) of the second vertical vein.

This report describes a successful intervention by catheterization in the treatment of a VV shunt of TAPVC.

Total anomalous pulmonary venous connection (TAPVC) constitutes 1%‐1.5% of congenital heart diseases in children. Four types have been identified based on where the pulmonary vein connects abnormally: supracardiac, cardiac, infracardiac, and mixed, where the anomalous connection occurs at 2 or more of the above levels.^1^, ^2^

In the 2 cases discussed in this report, a second vertical vein was noticed incidentally during routine follow‐up catheterization 1 year after the initial surgery.

It originated proximally from the ligated vertical vein and drained into the superior vena cava through the accessory hemiazygos vein.

It seemed that blood perfusion to a second vertical vein had increased due to disappearance of dominant vertical vein blood perfusion after the initial surgery.

In this report, we discuss the management of this rare case. Furthermore, this is the first reported case of a transcatheter closure of a second vertical vein in a mixed TAPVC.

## 2 CASE REPORT

### 2.1 Case 1

**FIGURE 1:**
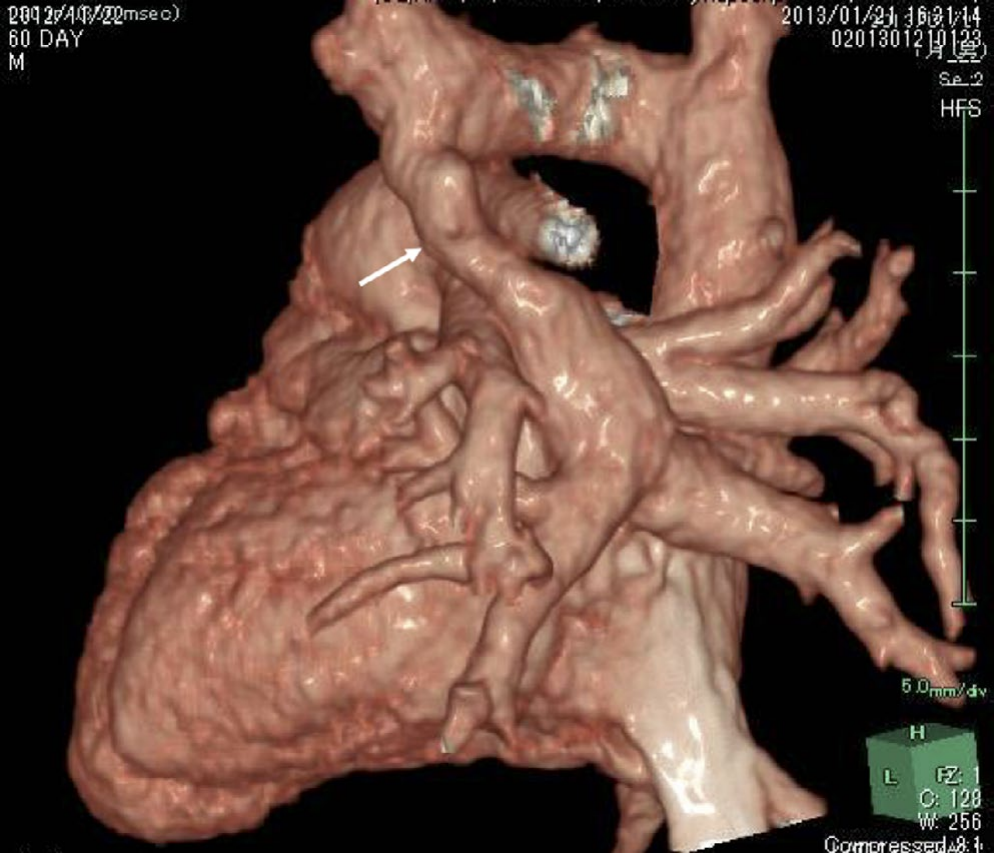
Contrast CT before surgery repair (behind view). White arrow indicates vertical vein (common PV to innominate vein)

A 9‐year‐old boy underwent surgical repair of supracardiac TAPVC when he was 2 months old and weighed 2.2 Kg [Figure 1].

**FIGURE 2:**
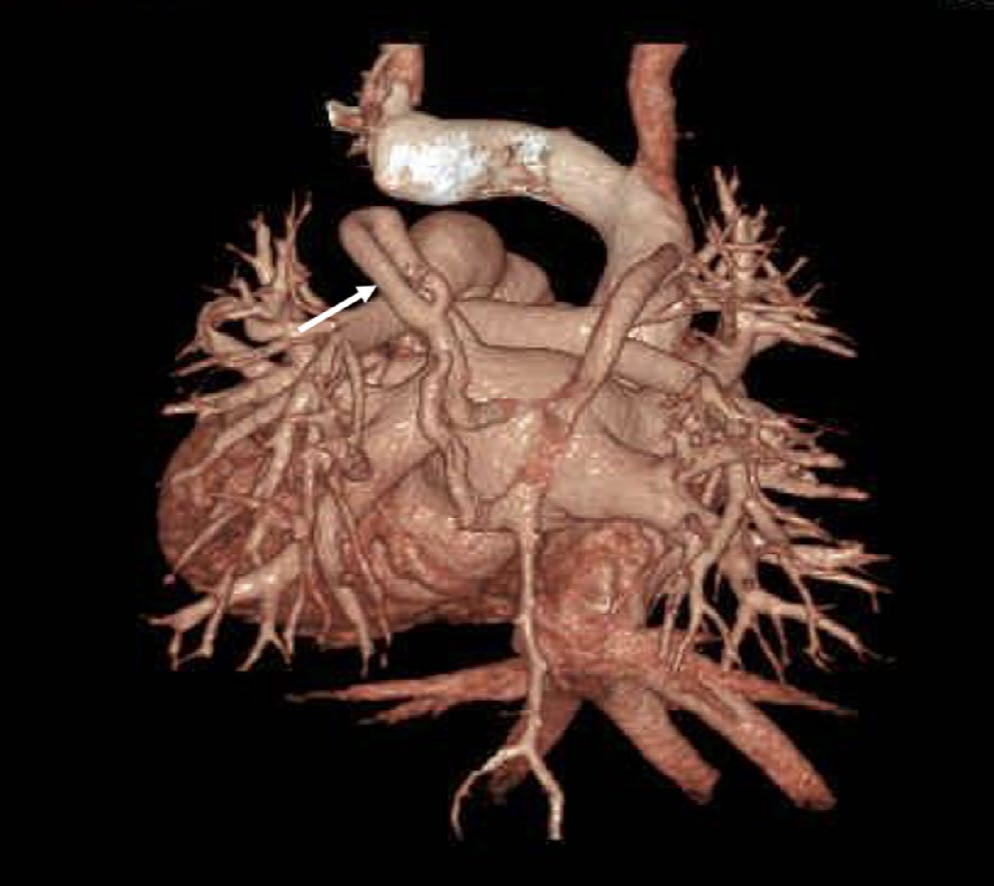
Contrast CT after surgery repair (behind view). White arrow indicates second vertical vein (originated from proximal ligated vertical vein and drain into superior vena cava)

During the operation, the vertical vein was ligated. One year after the surgical repair, we performed follow‐up diagnostic catheterization that showed obvious azygos vein enlargement. Contrast‐enhanced CT was performed, and it showed a veno‐venous shunt (VV shunt) that originated proximal to the ligated vertical vein and drained into the superior vena cava through the accessary hemiazygos vein‐azygos vein [Figure 2].

These findings showed there was a second vertical vein causing TAPVC. Therefore, we corrected the diagnosis from a supracardiac TAPVC to a double drainage of TAPVC, a rare variant of the mixed type. The left‐right shunting, which occurred as a result of this development, did not provoke right heart volume overload, and the general condition of the patient was good. His right heart function is under close monitoring in outpatient.

### 2.2 Case 2

A 2‐year‐old infant, weighing 9 kg, with a diagnosis of supracardiac TAPVC underwent surgical correction at 1 month. Preoperative echocardiography and contrast CT showed that the pulmonary veins formed a confluence and drained into the innominate vein through the vertical vein. We diagnosed supracardiac TAPVC without another cardiac anomaly.

During surgery, the vertical vein was ligated on the innominate vein side. Postoperatively, recovery was good and pulmonary venous stenosis (PVS) did not arise. Furthermore, using contrast CT, we confirmed that the connection of the common chamber and left atrium was not restrictive before the patient was discharged. A year after the surgical repair, we performed a follow‐up catheter examination to check the patients’ cardiac condition. It showed that central venous pressure was 5 mm Hg, right ventricular pressure was 23 mm Hg, mean pulmonary capillary wedge pressure was 9 mm Hg, left ventricular pressure was 63 mm Hg, left ventricular end‐diastolic volume was 104% of normal, and right ventricular end‐diastolic volume was 103% of normal.

**FIGURE 3:**
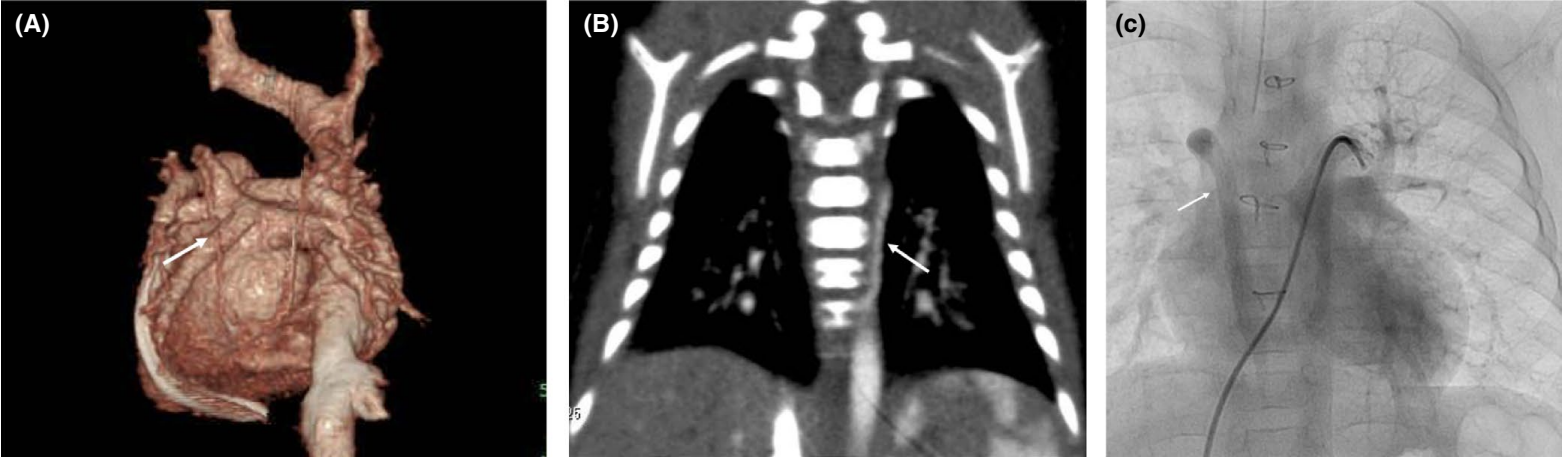
A, Contrast CT after surgery repair (behind view). White arrow indicates second vertical vein (originated from proximal ligated vertical vein and drain into superior vena cava). B, White arrow indicates accessory hemiazygos vein. C, Left pulmonary angiogram. White arrow indicates second vertical vein into superior vena cava

Although the patient was hemodynamically stable with no pulmonary vein stenosis (PVS), a second vertical vein was incidentally noticed that originated from the proximal ligated vertical vein through the accessary hemiazygos vein, which drained into the superior vena cava (Figure 3).

On initial assessment, it appeared as though the second vertical vein originated from the same location as the first vein. However, the first vertical vein was obviously dominant; therefore, the second vessel would have had much less perfusion and would have been difficult to be identified before surgery. After surgery, it became visible as it had not spontaneously atrophied but rather had increased perfusion. At the time of discovery, it appeared that the RV volume load was tolerable and would be managed by careful follow‐up. However, at the next check‐up, tricuspid regurgitation had gradually increased. The RV volume load appeared to be worsening, and we decided to perform occlusion of the second vertical vein.

**FIGURE 4:**
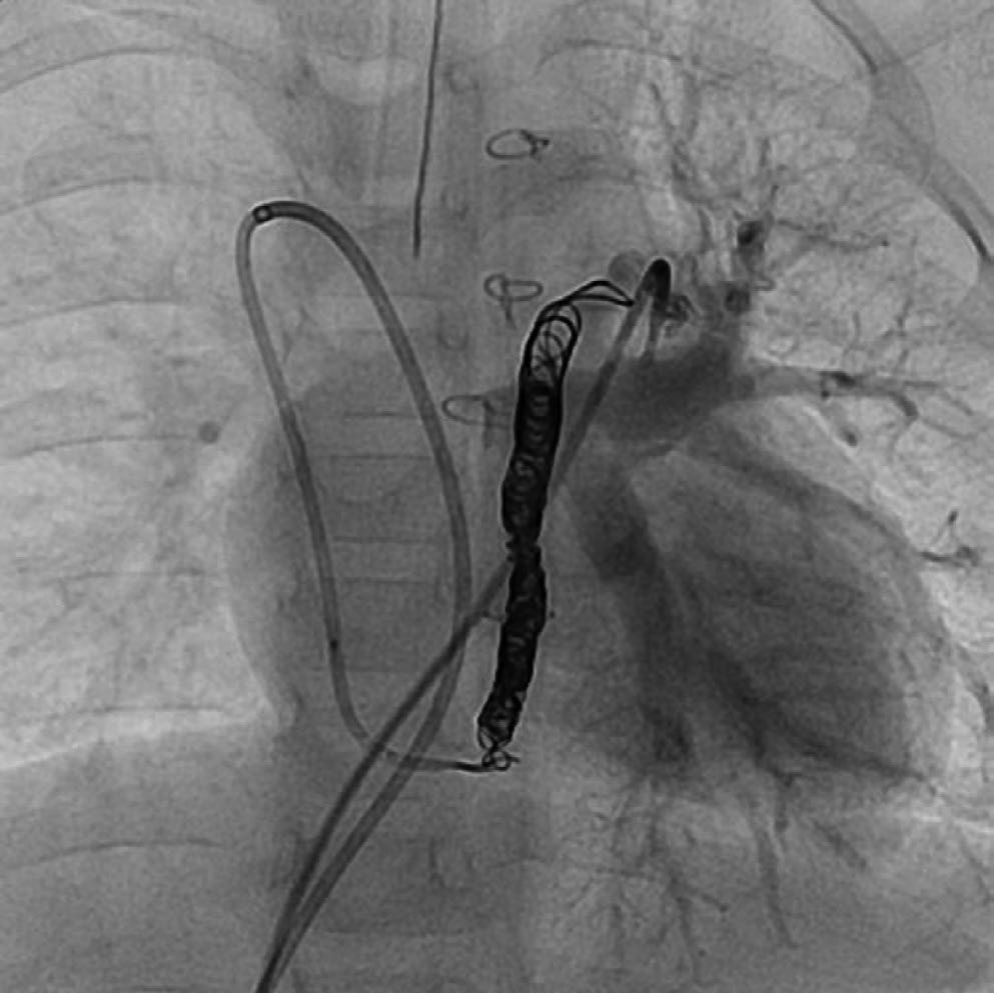
Left pulmonary angiogram after coil embolization

Next year of first catheter, we performed the treatment. The right femoral vein was accessed with a 5Fr venous sheath, and the accessary hemiazygos veins were accessed from the SVC using a 4 Fr/100 cm GlidecathⅡ catheter with a Berenstein tip (Terumo). A diagnostic contrast angiography using a 2.2 Fr Progreatβ^3^ microcatheter (Terumo) was also performed in the SVC and left pulmonary with occlusion 4Fr wedge in the azygos vein. The left pulmonary artery (LPA) mean pressure measured 22 mm Hg, showing an increase from the previous year. We carefully performed coil embolization to avoid occlusion of the pulmonary vein. The first coil (8 mm × 250 mm MICRUSFRAME^®^C coil; Johnson & Johnson) was introduced in close proximity to the origin of the VV shunt to avoid occlusion of the pulmonary vein, and angiography showed it was well positioned. Additional coils (6 mm × 250 mm, 5 mm × 200 mm, 4 × 150 mm DELTAFILL^®^; Johnson & Johnson) and a final one (3 mm × 100 mm AZUR^®^; Terumo) were placed to ensure complete occlusion (Figure 4). Repeat angiography showed disappearance of the residual flow from the VV shunt. Mean left pulmonary artery pressure measurements revealed the same result before and after the procedure, and the patient was discharged from the hospital 2 days later.

## 3 DISCUSSION

Double drainage of TAPVC is a rare variant of a mixed type TAPVC, which occurs when all the pulmonary veins form a confluence and then drain to both the coronary sinus and the left innominate vein.^3^, ^4^

Recently however, other variants of TAPVCs with double drainage have been reported.^5^, ^6^

In our 2 cases, the pulmonary veins formed a confluence and drained into the systemic venous system via two vertical veins. The first of the vertical veins drained through the innominate vein, and the second originated from the proximal side of the ascending vertical vein.

Preoperative identification of TAPVC with double drainage has important surgical implications. Although in both cases we performed echocardiography and contrast CT before the initial surgery so as not to miss the diagnosis, we did not detect this anomaly.

Because the first ascending vertical vein was more prominent and blood flowed without obstruction, the second vertical vein had less perfusion and a smaller vessel diameter. However, after the initial surgery, perfusion to the dominant vertical vein decreased, causing an increase in blood flow to the second vertical vein. Pulmonary venous obstruction, with or without confluence stenosis, is a well‐known complication occurring in approximately 8%‐15% of patients after surgical correction of TAPVC. ^7^

It was reported that with smaller left‐sided chambers and a noncompliant left atrium, an unligated vertical vein may improve survival by preventing a pulmonary hypertensive (PH) crisis.^8^

An unligated vertical vein has been reported to atrophy spontaneously.

However, if it remains patent, it may cause right cardiac failure due to left‐to‐right shunting.^9^

Pulmonary venous stenosis did not occur after surgery in either of the cases, and on follow‐up, using chest X‐rays and echocardiography for diagnostic imaging, this complication was not detected. In our institution, a follow‐up diagnostic catheterization for post‐TAPVC surgery patients is performed 1 year after the operation routinely. If this catheterization is not performed, this anomaly can go undetected. While echocardiography is sufficient for diagnosing most TAPVC cases, cardiac catheterization is essential in a mixed variety to adequately assess drainage and possible obstruction of all four pulmonary veins.^10^

Although cardiac catheterization and contrast CT are useful tools, it causes exposure of patients to ionizing radiation. Therefore, we should use echocardiography in routine follow‐up after TAPVC repair to avoid useless ionizing radiation, and it might be better perform these modalities when we detect features of unexplained left‐to‐right shunting.

We performed angiography and contrast CT again in another patient who underwent repair of a supracardiac TAPVC 10 years ago in our institution; this patient did not present with the same anomaly. The left‐to‐right shunting persisted, and we did not identify right heart volume overload early as was done in Case 1. The hemodynamics in this patient however mimicked an atrial septal defect, and the right heart volume load gradually increased as he became an adult.

On the other hand, in Case 2, there was significant right heart volume overload and it caused an exacerbation of the tricuspid regurgitation, resulting in an increase of the mean pulmonary artery pressure. Coil embolization of the second vertical vein was therefore appropriate in this case.

Previously, it was recommended that the vertical vein was deliberately not operated on in order to prevent a PH crisis, with embolization with a coil or plug to be performed if the right heart volume load increased.^11^, ^12^

If we could ligate vertical vein near the pulmonary vein, these results might not be occur.

To our knowledge, this is the first case of a coil embolization for a vessel that originated from the proximal ligated vertical vein and drained into the superior vena cava through an accessory hemiazygos vein‐azygos vein after a TAPVC repair.

Total anomalous pulmonary venous connection of mixed type is often misdiagnosed if left‐to‐right shunting is present like in our case. This procedure proved to be safe and provided an alternative noninvasive treatment that did not involve surgery.

## 4 CONCLUSION

We encountered two rare cases of mixed type TAPVC with double drainage, where the second vertical vein enlarged rather than atrophied after surgery. Echocardiography is a useful diagnostic tool in the follow‐up of PVS; however, contrast‐enhanced CT or diagnostic catheterization should be performed to exclude a second veno‐venous shunt postsurgery if we detect unexplained features of left‐to‐right shunting. Cases of left‐to‐right shunting that is not recognized before surgery can be treated with catheterization as demonstrated by our case.

## CONFLICT OF INTEREST

None of the authors have any financial or personal conflicts of interest.

## AUTHOR CONTRIBUTIONS

HN: curated the data; performed formal analysis; involved in investigation; provided software; visualized the data; and wrote the original draft. KT: involved in investigation; performed methodology; and provided resources. NK: involved in investigation; provided resources; and validated the data. TT: conceptualized the study; involved in project administration; supervised the study; and validated the data.

## ETHICAL APPROVAL

The study was published with written consent from parents of the patients.
